# Solvation Forces Near Hydrophobic Surfaces: A Classical Density Functional Theory Study

**DOI:** 10.1021/acs.jpcb.4c01426

**Published:** 2024-07-19

**Authors:** Simone Riva, Ofer Manor

**Affiliations:** Department of Chemical Engineering, Technion—Israel Institute of Technology, Haifa 3200003, Israel

## Abstract

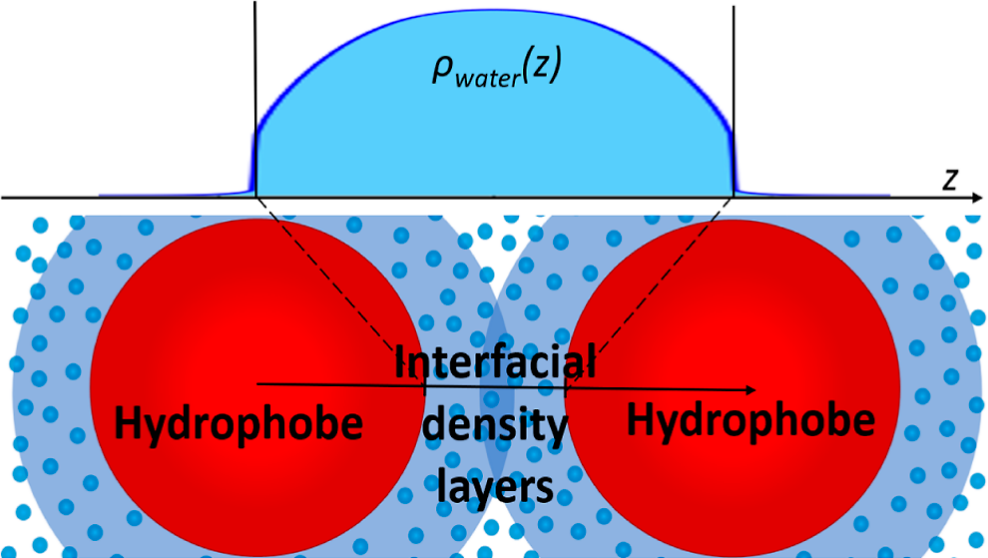

We use classical density functional theory (DFT) to model solvation interactions between hydrophobic surfaces, which we show to be characterized by depletion attraction at small surface to surface separations and a slowly decaying bipower law interaction at large separations. The solvation interaction originates from van der Waals (vdW) and Coulombic interactions between molecules in the polar solvent, e.g., water, and from the molecules thermal motion and finite volume. We investigate model hydrophobic surfaces represented by bubbles and nonpolar solids, e.g., aliphatic particles, and calculate in a DFT fashion the distribution of molecules in the interlaying solvent between two such surfaces and the hydrophobic excess force resulting from it. The interactions are largely attractive, which is well-known in measurement, albeit vdW attraction between molecules in solids and in the solvent may cause repulsion at certain interface to interface separations. We commence our analysis by suggesting an asymptotic analytical bipower law expression for the solvation interaction at large separations. Thereafter we present a full numerical solution, which is in good agreement with the analytical prediction and further explores the interaction at small surface to surface separations. Our theoretical results yield adhesion energies which agree with previous experiments.

## Introduction

Hydrophilic and hydrophobic forces have been treated as exponentially decaying interactions since the experimental observations by Israelachvili and Pashley^1^ and following the proposed unified-form potential which included both interactions.^2^ Although ubiquitous in many chemical and biological processes, a comprehensive understanding of hydrophobic forces is still lacking to date.^3^ A number of attempts were made to explain their origin^4^ using empirical models for fitting experimental measurements, albeit a definitive closed theoretical model has not been established. Various mechanisms were proposed as originating the hydrophobic interactions in different regimes. At least at short separation between interacting surfaces they are believed to be associated with solvent structuring effects near the surface, namely variations in water density and in water molecule orientation.

Several studies investigated hydrophobic interactions theoretically by means of density functional theory (DFT).^5−7^ A similar approach was adopted by Pismen et al. to evaluate the disjoining pressure in free liquid films near a three phase contact line.^8−10^ In these studies, the authors solve an integro-differential density field equation to calculate the dynamic density profile of the diffuse vapor/liquid interface of a liquid film atop a solid substrate to predict the corresponding static three phase contact angle and the dynamics of film spreading. Moreover, they study the disjoining pressure in the film due to van der Waals (vdW) molecular interactions and show an asymptotic representation of the disjoining pressure as a bipower law for sufficiently thick films.

**Figure 1:**
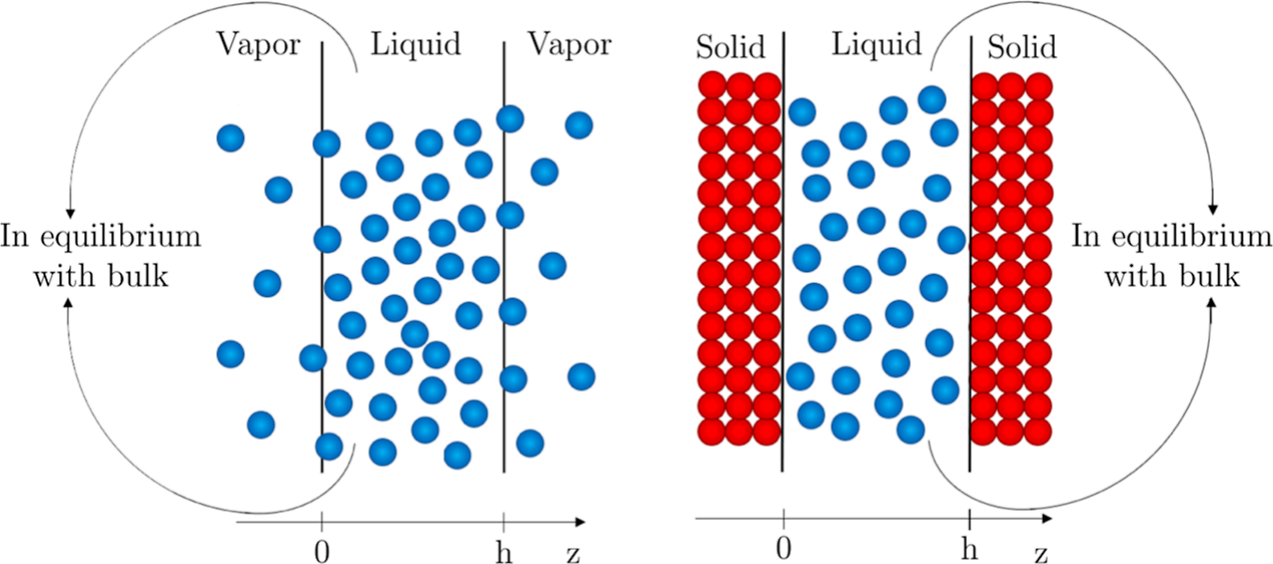
Illustration of the model for the interaction between two vapor/liquid and two solid/liquid interfaces, where water molecules interact with each other via entropic, vdW, and Coulombic interactions and with the solid via vdW interactions.

We use classical density functional theory to connect hydrophobic forces to interactions between bipolar molecules, such as water, and suggest a corresponding theory-based analytical model. We extend the studies by Pismen et al. to the interaction potential in a liquid film formed between two surfaces, either liquid–vapor or hydrophobic solid. Furthermore, the present theory of confined water expands the aforementioned procedure to account for vdW and Coulombic contributions to the uniform-phase free energy. Our model for the effective interaction between two particles is constituted by two infinite planar interfaces delimiting a polar liquid film—see Figure 1. Ultimately, we express this interaction in terms of the potential energy of the solvent and the force on interacting spherical particles via the Derjaguin approximation, which aligns with a range of measurements.

## Theoretical Methods

### Vapor Interfaces

We commence our analysis by calculating the hydrophobic interaction between two vapor bubbles, namely two interfaces that separate a dilute (vapor) phase from a dense (liquid) phase; this is a benchmark for hydrophobic surfaces when the phases are associated with water. The vapor and liquid phases are assumed at thermodynamic equilibrium, which supports stable interfaces. This means that at every fixed temperature, *T*, the chemical potential is chosen as the liquid–vapor coexistence chemical potential, μ_eq_(*T*). Hence, the corresponding vapor and liquid bulk densities, ρ_v_ and ρ_l_, are associated with minima in the Landau free energy; the equilibrium chemical potential, μ, fulfills the Maxwell construction, thus the energy minima are of equal depth. The Maxwell construction provides a practical way of calculating μ as the value that equalizes the bulk free energies of the two phases, prescribed in our theory.

The Landau free energy, , is given by the difference between the Helmholtz free energy, , and the chemical potential contribution, μ*N*, where we calculate the total number of particles, *N*, using the integral of density. Given the Helmholtz free energy per molecule, *f*(ρ), of a uniform phase of density ρ, the Landau free energy is further given by the volume integrals^11,12^
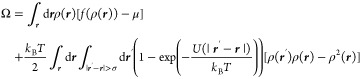
1where ***r*** and ***r*′** are vectors of a spatial coordinate system, ρ(***r***) is the liquid density, σ is the diameter of the solvent molecules assuming they are hard spheres, *k*_B_*T* is the thermal energy, and *U* is the isotropic intermolecular interaction potential. The first integral on the right-hand side of the equality gives the Landau energy for a locally uniform liquid. The second integral accounts for nonlocal excess mean-field contributions to the free energy when the liquid density is nonuniform.^13^ In our analysis we consider an intermolecular interaction potential due to vdW forces, *U*(*r*) = −*C*_11_/*r*^6^ at *r* > σ, while assuming the solvent molecules possess a hard-core diameter, σ, so that *U*(*r*) → ∞ at *r* < σ, where *r* ≡ |***r′*** – ***r***|. The subscript 11 refers to vdW interactions between two solvent molecules.

At uniform density, ρ, the Helmholtz energy per unit volume of a vdW fluid is , with  and *a* = −2*πk*_B_*T*∫_σ_^∞^d*r*[1 – exp(−*U*(*r*)/*k*_B_*T*)]*r*^2^, in the mean-field approximation.^14^ Assuming a polar solvent, e.g., water, we further account for the electrostatic energy between solvent molecules by approximating the molecules in the form of dipolar hard spheres and further using the random phase approximation, suggested elsewhere,^15−17^ to obtain the Helmholtz free energy of correlated dipolar spheres, given by *ρf*_el_(ρ) = −(*k*_B_*T*/*l*^3^)(1 – 3α/4)*m*(*y*); in this approximation, *y* = ρ*p*^2^/3ε_0_*k*_B_*T*, *p* ≡ *ql* = 1.85 D being the dipolar moment of water molecules, *q* the effective charge difference along the molecule, and *l* the dipole length; moreover, α = η(4 – η)(2 + η^2^)/(1 + 2η)^2^ is the solvent compressibility factor in the Percus–Yevick approximation, wherein η = (π/6)σ^3^ρ is the molecules volume fraction, and . While the model for electrostatic interactions between the polar solvent molecules does not explicitly treat hydrogen bond structural effects, ignoring the asymmetry in the interaction while assuming isotropic spherical mass of molecules, it is a leading order approximation thereof. We thus employ both vdW and electrostatic (or Coulombic) contributions to the Helmholtz free energy, that at uniform density, ρ, is given per molecule by *f*(ρ) = *f*_vdW_(ρ) + *f*_el_(ρ).

Equation 1 contains the Mayer function *f*(*r*) = exp(−*U*(*r*)/*k*_B_*T*) – 1, which expresses the deviation from ideality in the nonlocal interaction. We write the Landau energy per unit surface area, *A*, as the integral^18^
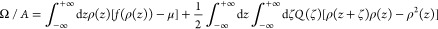
2where we approximate the Mayer function to the linear order by *f*(*r*) ≈ −*U*(*r*)/*k*_B_*T* and use planar Cartesian symmetry between the interacting surfaces, assuming two planar surfaces parallel to the *x* – *y* plane and positioned at *z* = 0 and *z* = *h*. The interaction kernel
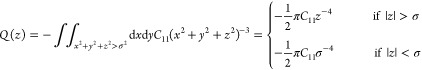
is the total vdW interaction energy between a molecule and a plane perpendicular to the relevant *z* coordinate at constant unitary density. This reduces the inner integral dimensions to one in terms of *Q*(*z*), as the water density is constant over such planes.

The equilibrium solvent density profile ρ(*z*) is given by minimizing the free energy
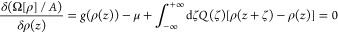
3namely equating its functional derivative to zero, where *g*(ρ) ≡ d[ρ*f*(ρ)]/dρ. Solving eq 3 leads to the equilibrium density distribution ρ(*z*; *h*) that varies along the coordinate *z* between the parallel surfaces; the density depends parametrically on the surface to surface separation, *h*. We obtain the corresponding interaction energy per unit surface area by substituting ρ(*z*; *h*) in Ω(*h*)/*A*. We further use the Derjaguin approximation, which provides a method to obtain the force between curved objects from the interaction energy of planar surfaces, provided that their minimum distance is much smaller than their size. The force between identical spheres normalized by their radius *R* reads *F*(*h*)/*R* ≈ πΩ(*h*)/*A*. In the following the results are presented in terms of force *F*(*h*)/*R* between spheres, but the corresponding energy per unit area Ω(*h*)/*A* between planar surfaces is also reported in all graphs on the right vertical axis.

### Solid Surfaces

Further considering solvation interaction between hydrophobic solid surfaces, e.g., aliphatic surfaces, we replace the vapor bubbles with solid spherical particles. The solid particles confine solvent molecules within the interlaying solvent film. We commence our analysis by assuming two flat solid objects interacting through a water film. The flat solid/water planes are placed at *z* = 0 and *z* = *h* using the perpendicular coordinate *z*. Here we introduce the no-penetration constraint of water molecules into the solid. Hence, the solvation interaction energy is given by spatial integrals limited between the two solid/water planes. We further add vdW contributions produced by interactions between the molecules in the solids and water as an external potential *V*_ext_. The corresponding grand potential for the interaction energy, Ω, per unit area, *A*, is thus
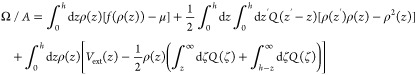
4where the external potential is the sum of two single-wall potentials and thus is given by *V*_ext_(*z*) = *w*(*z*) + *w*(*h* – *z*). The second term in the third integral is necessary to remove the homogeneous liquid interactions with the volume now occupied by the particles, which are contained in the parameter *a* in the free energy *f*. It cancels exactly the term proportional to *a* in the first integral and the term proportional to ρ^2^(*z*) in the double integral, so that this equation is reduced to
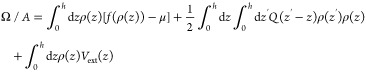
5where we redefined the function *f*, so that ρ*f*_vdW_(ρ) ≈ *k*_B_*T*ρ ln(ρ/(1 – *b*ρ)). Furthermore, we assume that the solvent molecules and the aliphatic solid interact via vdW forces, labeled by the subscript “12”, so that the single-wall potential is given by
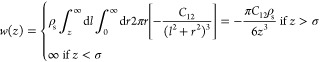
6and the Euler–Lagrange equation that corresponds to the new functional is given by
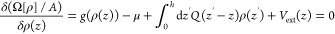
7

### vdW Parameters and Hamaker Constants

In order to estimate the parameters of vdW theory, especially *b*, which is the natural volume unit of the system, we resorted to the vdW theory of condensation.^19^ According to it, the parameter *b* is related to the critical constants by . From the known critical temperature and pressure of water it is trivial to find *b* = 5.05 × 10^–29^ m^3^, which corresponds to σ = 2.89 Å. Moreover, from vdW theory *a* = 27*b*^2^*p*_c_. From the definitions *a* = −2π*k*_B_*T*∫_σ_^∞^d*r*[1 – exp(−*U*(*r*)/*k*_B_*T*)]*r*^2^ and , approximating linearly the Mayer function in the former, one finds , thus the constant *C*_11_ of dispersion interactions in water can be determined: *C*_11_ = 1.75 × 10^–77^ J m^–6^. This constant is related to the Hamaker constant of the liquid through *A*_11_ = π^2^*C*_11_ρ_l_^2^ = 4.90 × 10^–20^ J at room temperature. This is close to the value calculated based on the Lifshitz theory of vdW forces in water: *A*_11_ = 3.7 × 10^–20^ J.^20^

In the solid case, we undertook calculations for different intensities of the vdW attraction between solid and water molecules, which is characterized by the Hamaker constant *A*_12_, scaled by the liquid-liquid one *A*_11_. The latter was given above, while the former is *A*_12_ = π^2^*C*_12_ρ_l_ρ_s_, with ρ_s_ the solid density. We define the dimensionless quantity, , which reveals that the fundamental parameter is the ratio between *C*_12_ρ_s_ and *C*_11_ρ_l_. The Hamaker constant for the solid particles interacting through vacuum is *A*_22_ = *H*^2^*A*_11_, in order to fulfill the Berthelot rule.^21^ In the case of *H* = 1.1, one of the values we used in the calculations below, *A*_22_ is approximately 6 × 10^–20^ J, thus corresponding to the range of Hamaker constants measured for silica particles in vacuum and being close to their theoretical value.^20^

### Computational Methods

The Euler–Lagrange equation to minimize the Landau free energy functional was solved numerically through an iterative algorithm implemented in Julia.^22^ In both cases, bubbles and solids, a nonlinear integral equation of the second kind has to be solved—see eqs 3 and 7. We made use of the successive approximation method or Picard method.^23,24^ In each iteration, the algebraic equation for ρ(*z*) is solved for a set of *z*, given an estimate of the integral based on the previous step.

**Figure 2:**
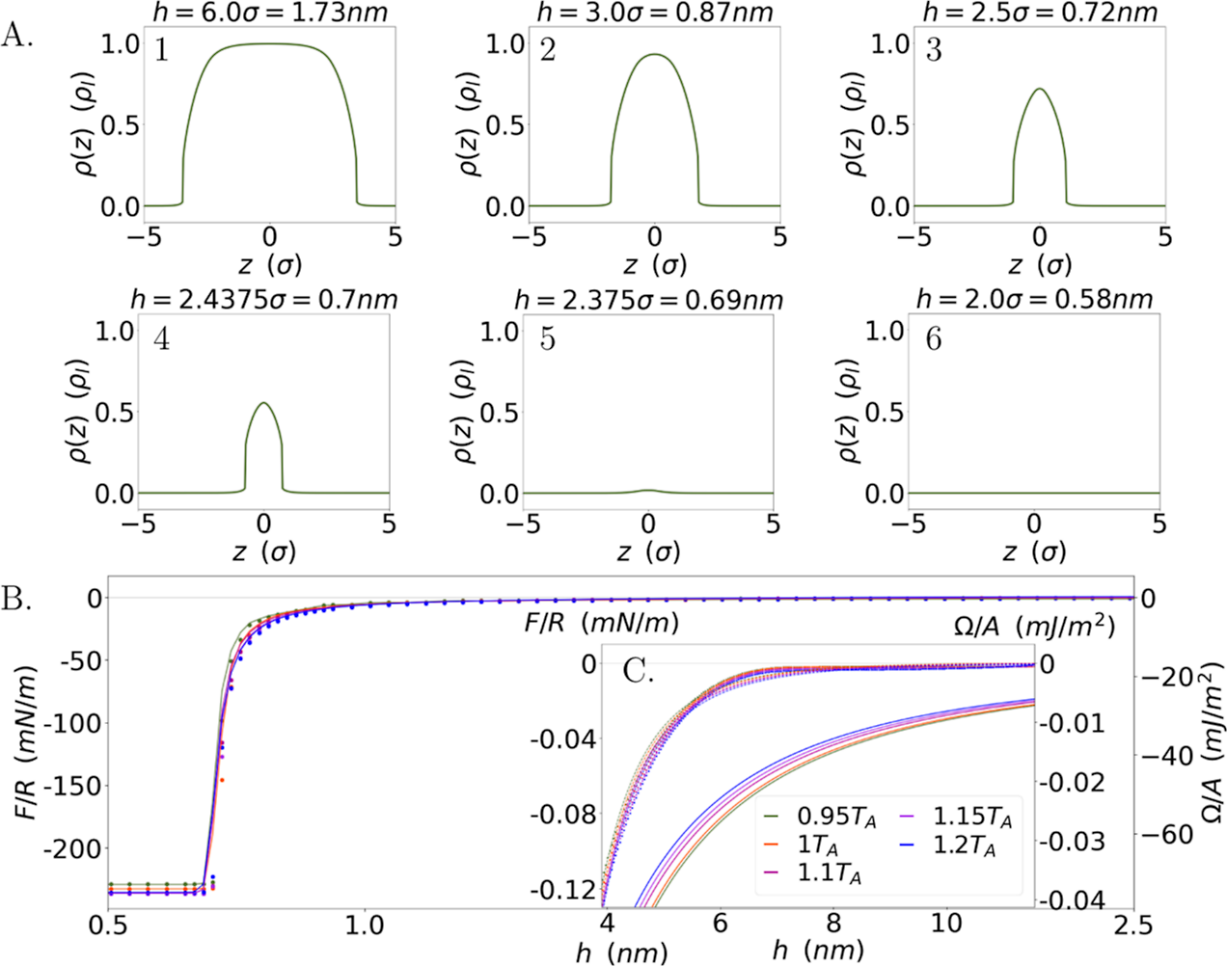
(A) Spatial solvent density distributions in a film between two *h*-separated flat vapor/liquid interfaces, where length units along the *z* axis are the solvent molecule diameter, σ, and density units are the bulk density ρ_l_. (B) Separation (*h*) variations of the interaction force (energy on the right vertical axis) between bubbles (flat vapor/liquid interfaces) at different temperatures, where points give numerical results for the interaction force (energy) in the temperature range (given by curves at different colors) between water freezing and boiling at atmospheric pressure; solid lines are a guide to the eye. (C) In the inset we show the large separation decay of the interaction, where points give numerical results, dashed lines give the best fit of numerical results with the function α_0_ + α_1_*h*^–2^ + α_2_*h*^–5^ and solid lines give analytical results.

**Figure 3:**
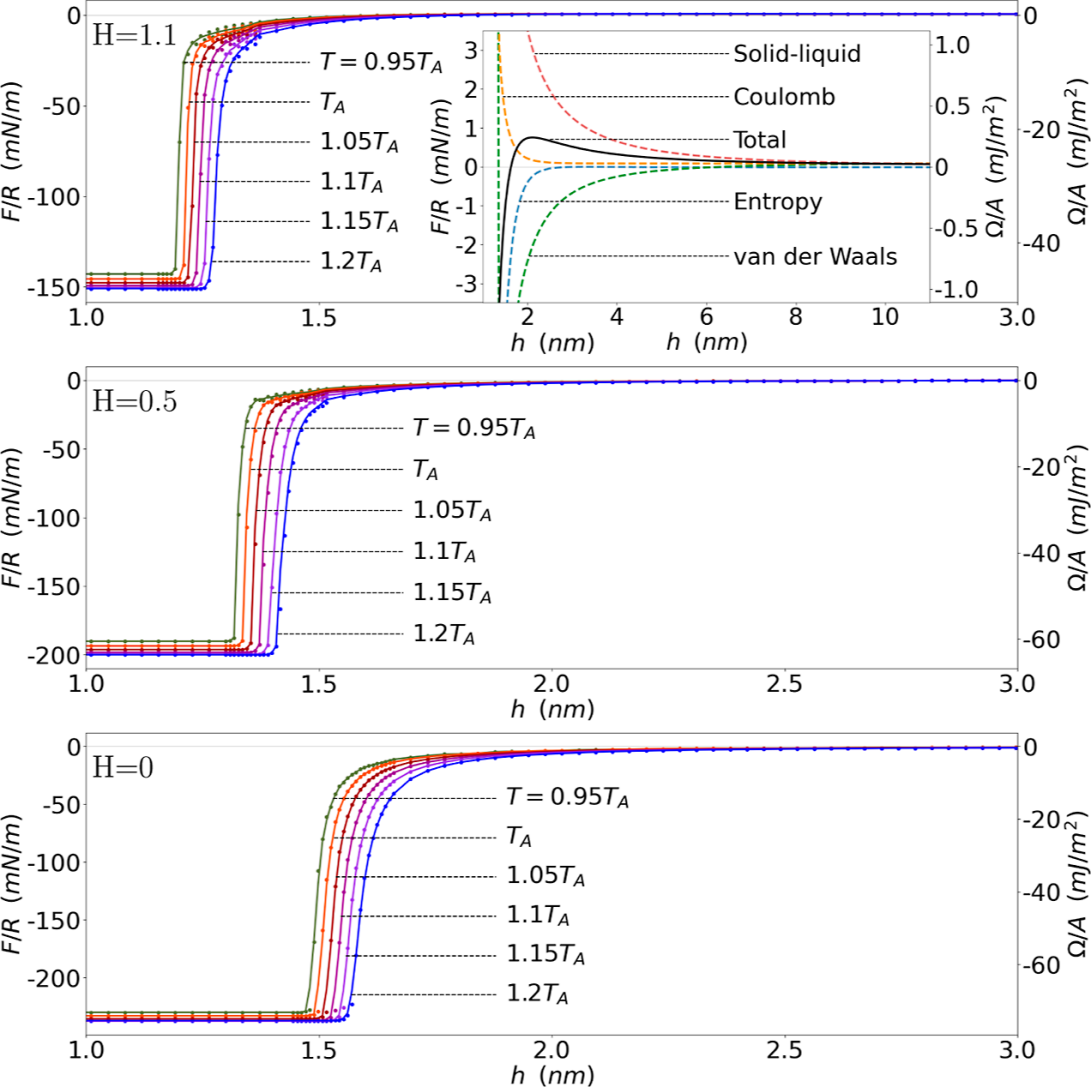
Separation (*h*) variations of the interaction force (energy) between hydrophobic spherical solids (flat solid/solvent interfaces) for different solvent–solvent and solid–solvent Hamaker constant ratios: *H* = 1.1, 0.5, 0, where points are numerical results and lines are a guide to the eye. Inset: *h* variations of different contributions to the interaction energy and their superposition (in black) for *H* = 1.1, *T* = 0.95*T*_A_. “Entropy” refers to the term *k*_B_*T*∫_0_^*h*^d*z*ρ(*z*)ln[ρ(*z*)/(1 – *b*ρ(*z*))], “van der Waals” to ∫_0_^*h*^d*z*ρ(*z*)∫_0_^*h*^d*z*′*Q*(*z*′ – *z*)ρ(*z*′), “Coulomb” to ∫_0_^*h*^d*z*ρ(*z*)*f*_el_(ρ(*z*)) and “Solid–liquid” to ∫_0_^*h*^d*z*ρ(*z*)*V*_ext_(*z*) in eq 5.

Integrals calculation is performed using Simpson quadrature and is the primary source of numerical error. Errors from root-finding can be reduced by sampling the density with denser points using a simple bisection algorithm. This part of the calculation, being the least time-consuming, does not significantly impact performance since it scales linearly with the number of points. On the other hand, the quadrature consumes most of the computation time and scales quadratically with the number of *z* nodes, since the integrals are nonlocal. For this reason it is necessary to carefully choose the appropriate density of nodes in order to achieve balance between precision and computation time. The uncertainty is estimated by calculating the two-norm and infinity-norm of the solvent density difference between the last two iterations. The nodes’ density was selected to keep the former below 1% and the latter below 0.1%. This corresponds to an error of 0.1 mN/m in the calculated force. This requirement was satisfied everywhere excluding the narrow 0.1 nm gap in the separation range (*h*), where the interaction energy abruptly plummets, undergoing a sharp transition from the large separation limit where the interaction energy is approximately 0 to −10 mJ/m^2^ to the depletion regime, where the interaction energy is approximately −150 to −250 mJ/m^2^ in our computations. In this specific narrow range of separations, see Figures 2 and 3, the sudden density variation and the corresponding steep force gradient give rise to relative numerical uncertainty that may reach 40 and sometimes 50%. However, the numerical uncertainty associated with this separation region does not significantly modify the shape of the force curves plotted in the figures due to the large slope of the nearly vertical curves in this case. More importantly, our main result is the functionality of the interaction energy decay at large separations, where the errors are well below 1%. In addition to the numerical node density, the integral cutoff and the number of iterations were chosen according to the aforementioned error thresholds. In particular, 10 iterations and 32 nodes per molecular diameter σ were used in the calculations. The maximum separation considered was *h* = 40σ. In the bubble case, eq 3 was solved on the interval −15σ < *z* < 55σ, extending at least 15 molecular diameters into both surfaces, whose limits also correspond to the integral’s cutoff. The integral is calculated over an interval of ζ of the same size, 70σ, centered at 0; at distances greater than 35σ the integrand is suppressed enough by the function *Q* to be negligible. For *z* ≠ 0, the density outside of −15σ < *z* +ζ < 55σ is approximated with the density at the closest extreme. This corresponds to taking the density at a minimum distance of 15σ from the surface as the density far inside the bubble, which is justified by density variations being extremely small at this point.

In the solid case, the integrals are only calculated over the interval from 0 to *h*, so *z* and *z*′ are limited to it. The energy contribution from the surface is included through the external potential.

## Results and Discussion

### Vapor Interfaces

Given eqs 2 and 3, we first look for an analytical approximation for the interaction energy Ω(*h*) at large separation between benchmark vapor/water hydrophobic surfaces—i. e., assuming two interacting bubbles. The analysis, that further accounts for molecules distribution across the water/vapor interfaces, is detailed in the Supporting Information. The interaction energy between the two flat water/vapor interfaces is given by
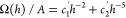
8where the powers of the separation *h* are products of the long-range vdW interactions and the coefficients *c*_1_′ and *c*_2_′ are products of the short-range Coulombic interactions between molecules alongside additional contributions from enthalpy and vdW interactions. Moreover, the low power component is associated with the integral of the vdW interaction—notice that *Q*(*z*) in eq 2 decays as *z*^–4^ and is integrated twice—and the high power component is associated with a further *h*^–3^ decay of the density away from an interface; the expressions for the coefficients are given in the Supporting Information.

Previously Dietrich^25^ and Schick^26^ calculated the surface free energy of an adsorbed finite liquid film on a wetting surface (rather than interacting drying surfaces as here) based on long-range vdW forces in the sharp-kink approximation. Thus, they assumed step-like profiles and ignored the shape of density variations and thus structural effects. In their work, similarly to ours, a leading-order *h*^–2^ term appears from nonretarded vdW interactions. They use elaborated power series to represent vdW interactions between molecules and molecules and continuum solids, hence they obtain additional powers of *h* such as −3, −4, etc. However, we account solely for the first term in the representation of vdW interaction between molecules, i.e., *U*(*r*) = −*C*_11_/*r*^6^ at *r* > σ, which allows us to analytically compute the free energy decay for the proper density profiles and the values of the coefficients. Hence, the successive correction in the present theory is constituted by a *h*^–5^ power, where a –2 contribution to the exponent is obtained from the previous leading-order approximations of the vdW interactions and an additional −3 contribution to the exponent is given from the correction to the density, which approaches the bulk value like *h*^–3^.

Using the Derjaguin approximation, the solvation force between two bubbles at close proximity reads
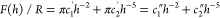
9The most striking feature of eq 9 is that the solvation force, hydrophobic force in this case, decays as a power law of the separation distance *h* between bubbles; the coefficients in the Supporting Information are given using complicated expressions, but the decay exponents are well-defined. Similar power-law decay at large separation is found in the numerical solution below.

The complete numerical solution of eq 3 is determined through an iterative algorithm, to which the separation *h* enters as a parameter. We obtain density profiles that satisfy equilibrium and, upon substituting them in eq 2, we calculate the interaction energy per unit area Ω/*A* as a function of the separation *h*. Figure 2 shows examples for spatial variations in water density with separation *h*, using water parameters in our analysis. The figure further shows the resulting diagram of the interaction energy and solvation force between vapor bubbles at different temperatures. The temperatures, relative to room temperature, *T*_A_ = 298 K, span from the freezing to the boiling point of water at atmospheric pressure, *T*_freeze_/*T*_A_ = 0.92 and *T*_boil_/*T*_A_ = 1.25. No significant difference is found between the curves at different temperatures for vapor bubbles.

Starting from large separations, the density initially displays two isolated and independent interfaces, marked by steep variations—see Figure 2A(1). At large separation, *h*, the density, ρ, at the center of the water film is close to the water bulk density, ρ_l_; in the vapor phase (in the bubbles) the scaled water density tends to ρ_v_ ≈ 2 × 10^–4^ρ_l_. The interaction force initially increases slowly away from nothing upon reducing *h*. Shortly before reaching a separation of *h* ≈ 3σ ≈ 9 Å the two interfaces begin to interfere significantly. As a result, the maximum density is reduced—see Figure 2A(2). We observe a rapid drop in solvent density at *h* ≈ 2.5σ ≈ 7.5 Å across the film, see Figure 2A(3–5), which corresponds to a sudden increase in the negative attractive force, due to the depletion of molecules in the film between the interacting surfaces. The density eventually flattens near *h* ≈ 2σ ≈ 6 Å, where the deviation of the water density in the intervening film from the vapor density becomes negligible, see Figure 2A(6). Here the whole system may be regarded as a pure vapor phase, i.e., the two bubbles are separated by a vapor phase and hence have effectively coalesced.

The interaction energy per unit area is constant at small separations, when the water concentration reaches the approximate value of water vapor, retaining mainly entropic (depletion) effects as the vdW and Coulombic interactions between the low density water molecules become small. Hence, requiring that the interaction energy vanishes at large separations renders attraction at small separations due to the depletion of water molecules. The Derjaguin approximation for the corresponding force between two spherical bubbles gives a vanishing interaction force at large separations and a constant attractive force at small separations.

The inset C in Figure 2 illustrates the solvation force decay at large separations (*h* > 4 nm). Inspired by our analytical findings—see eq 9—we further plot functions of the form *F*(*h*)/R = α_0_ + α_1_*h*^–2^ + α_2_*h*^–5^, with the coefficients α_0_, α_1_, α_2_ obtained by least-square fit with the numerical results in the separation range *h* = 4–12 nm, as dashed lines. The fitting functions describe rather accurately the numerical results. The analytical result subject to the theoretical predictions in Table 1 to the coefficients of eq 9 for the different cases are also given as solid lines for comparison and show qualitative agreement with the numerical results within the separation range employed in the simulation.

**Table 1:** Coefficients *c*_1_″ and *c*_2_″ for Interacting Bubbles in Water According to eq S4 in the Supporting Information and 9

*T*/*T*_A_	0.95	1	1.05	1.1	1.15	1.2
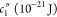	–4.775	–4.658	–4.527	–4.382	–4.220	–4.040
*c*_2_″ (10^–49^Jm^3^)	–11.242	–13.611	–16.079	–19.077	–22.522	–26.855

### Solid Surfaces

In the case of solid walls confining the liquid, the large separation analytical result for the solvation force is qualitatively similar, albeit the coefficients of the bipower law change. See the Supporting Information for the complete expressions.

We further solve eqs 7 and 5 numerically and obtain qualitatively similar insights to the previous case. We observe depletion of water molecules at separations smaller than *h* ≈ 4.5σ ≈ 13.5 Å. This is compared to *h* ≈ 2.5σ ≈ 7.5 Å in the case of vapor/water interfaces. The difference between the two cases is that molecules cross the vapor/water interface but not the solid/water interface. In the latter case, the molecule center may reach a minimum distance of about one diameter from the solid boundary, positioning the solid surface at the center of the solid molecules at its boundary and assuming solid molecules with size similar to the solvent, i.e., water. The density depletion in this case reveals a drying of the hydrophobic surfaces upon reaching a certain proximity, which is associated with the strong attractive force.

Figure 3 shows the behavior of the solvation force at different temperatures for various ratios between vdW interactions between water molecules and between water and solid molecules, *H* ≡ *A*_12_/*A*_11_ = *C*_12_ρ_s_/*C*_11_ρ_l_. Moreover, the different contributions to the total energy (or force) for *H* = 1.1 and *T* = 0.95*T*_A_ are shown in the inset, where we highlight the significance of both vdW and Coulombic interactions and the presence of a repulsive force maximum to result from *H* > 1.

**Figure 4:**
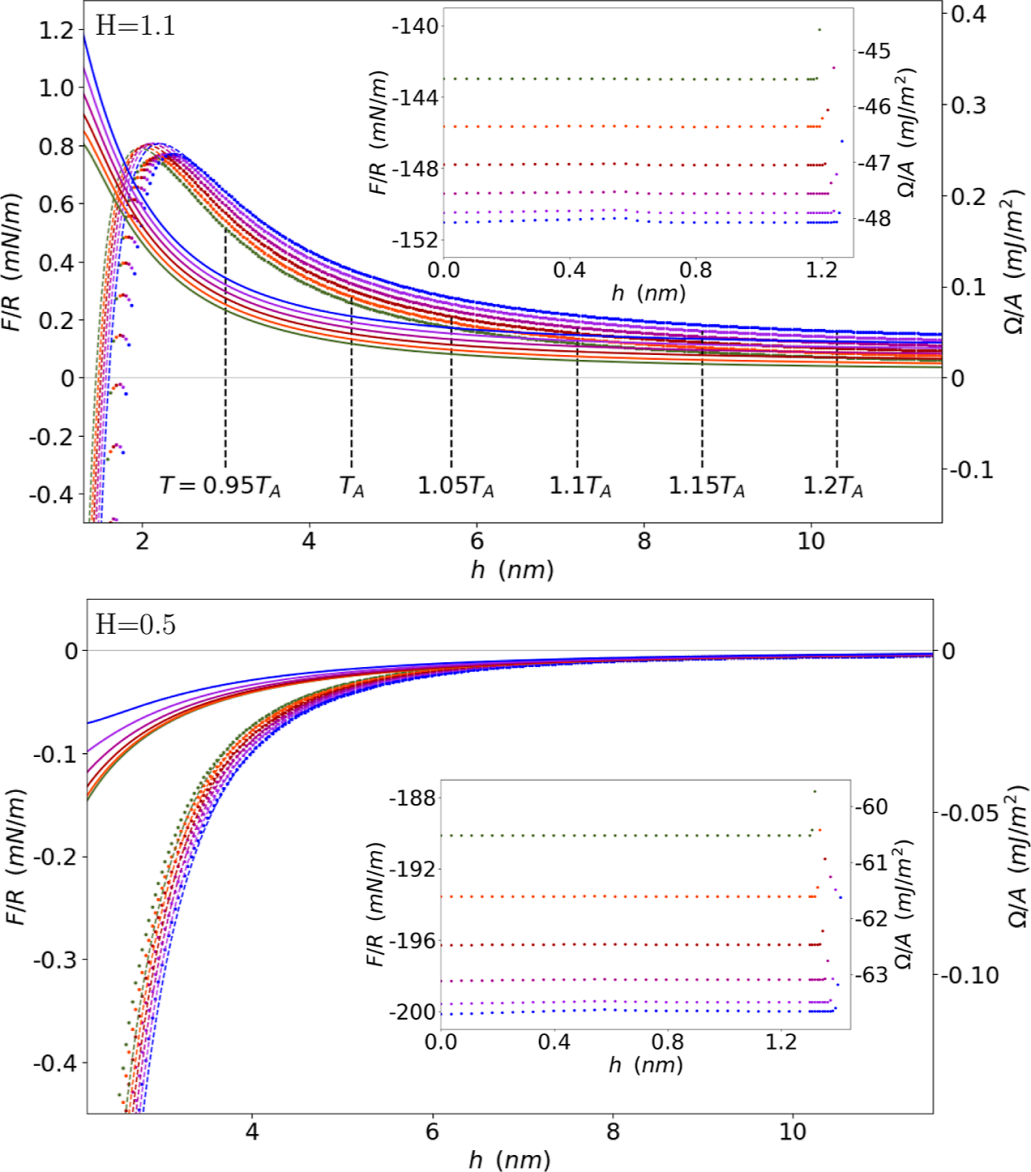
Large separation (*h*) variations of force (energy) between solids for different solvent–solvent and solid-solvent Hamaker constant ratios: *H* = 1.1 and 0.5, where points give numerical results for the interaction force (energy) in the temperature range, dashed lines give best fit of the numerical results with the function α_0_ + α_1_*h*^–2^ + α_2_*h*^–5^, and solid lines give analytical results. Insets: *h* variations of the attractive depletion force (energy) at small *h*.

In Figure 4 we present details of short and long-range behavior of the curves for vdW strength ratios, *H*, that are larger and smaller than 1. The insets highlight the attractive depletion force. As in the bubble interaction case, we observe that the analytical result for the interaction force obtained for large separation, *F*(*h*)/*R* = *c*_1_″*h*^–2^ + *c*_2_″*h*^–5^, qualitatively agrees with the simulation for separations from *h* = 2 nm to *h* = 12 nm. We give the corresponding coefficients in Tables 2 and 3 for *H* = 1.1 and *H* = 0.5 respectively. Moreover, while at *H* = 0.5, 0, the interaction is always attractive, at *H* = 1.1 the interaction exhibits a repulsive energy maximum, reflected by the positive sign of *c*_1_″. This maximum is therefore predicted by both our analytical result and numerical solution, and appears because water molecules experience a stronger vdW attraction from the solid than from the liquid bulk, i.e., *H* = *C*_12_ρ_s_/*C*_11_ρ_l_ > 1. The repulsive interaction may be regarded as a hydrophilic-like behavior of the solid surfaces. Overall, we obtain quantitative agreement between the same bipower law and the simulated force, excluding at small separations, by using least-square fit to obtain alternative coefficients. This functional dependence, that was encountered both in interactions between vapor bubbles and between solids, appears to be fundamental to the solvation interaction in our analysis.

**Table 2:** Coefficients *c*_1_″ and *c*_2_″ for Interacting Solids in Water According to eq S6 in the Supporting Information and 9; *H* = 1.1

*T*/*T*_A_	0.95	1	1.05	1.1	1.15	1.2
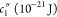	2.056	2.012	1.966	1.919	1.869	1.817
*c*_2_″ (10^–49^ Jm^3^)	–14.474	–13.865	–12.832	–11.346	–9.281	–6.561

**Table 3:** Coefficients *c*_1_″ and *c*_2_″ for Interacting Solids in Water According to eq S6 in the Supporting Information and 9; *H* = 0.5

*T*/*T*_A_	0.95	1	1.05	1.1	1.15	1.2
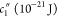	–0.687	–0.642	–0.601	–0.563	–0.528	–0.495
*c*_2_″ (10^–49^ Jm^3^)	–3.883	–2.006	0.494	3.757	7.936	13.246

### Comparison with Measured Hydrophobic Interactions

Solvation forces and in particular hydrophobic forces have been measured using different techniques,^27^ e.g., with the surface force apparatus (SFA) since the 1980s,^28^ which was followed by atomic force microscopy (AFM).^29,30^ Albeit, the interaction was usually modeled empirically as an exponentially decaying force, e.g., *W*(*h*) = −*W*_0_ exp(−*h*/*D*_H_), where *W*_0_ is indicative of the magnitude of the force and *D*_H_ gives the characteristic decay length of the force, experimental measures and their typical magnitudes are comparable to the range of interaction energy at contact (small *h*) in the present work, where we consider water as solvent. We found an energy of approximately −70 mJ/m^2^ for bubbles at all temperatures considered, while it ranges form −45 to −48 mJ/m^2^ and form −60 to −64 mJ/m^2^ at different temperatures for solids having a Hamaker constant *H* = 1.1 and 0.5 respectively. Experiments on cetyltrimethylammonium monolayers and poly(dimethylsiloxane), for instance, found that *W*_0_ = 22 mJ/m^2^, *D*_H_ = 1 nm and *W*_0_ = 94 mJ/m^2^, *D*_H_ = 1.7 nm, respectively.^2^ For mica/water/air interfaces *W*_0_ = 21 mJ/m^2^, *D*_H_ = 0.8 nm in one case and *W*_0_ = 72 mJ/m^2^, *D*_H_ = 1 nm in another case, the two cases differing by hydrophobicity levels, which correspond to water/solid/vapor three phase contact angles of 45 and 90°, respectively.^31^ Hence, our theory predicts similar order of magnitude of the adhesion hydrophobic energy to the corresponding magnitudes of *W*_0_ measured in experiments.

## Conclusions

In summary, we extended the studies by Pismen et al. to the interaction potential in a liquid film between two vapor/liquid or hydrophobic solid surfaces^32^ to study the hydrophobic force law. Making use of classical DFT,^33,34^ we presented a semianalytical derivation of the local density function of a fluid medium subject to vdW^35^ and Coulombic interactions. We wrote a Landau free energy functional describing the effective interaction between two flat surfaces of either vapor bubbles or solids through a film of a polar liquid, e.g., water; by using the Derjaguin approximation it is straightforward to translate this analysis to the interaction between nonplanar objects at small proximity.

We gave insight into the solvent mediated interaction between the two surfaces,^36^ particularly for large surface to surface separations (with respect to the solvent molecule size), where we derived an analytical result: the interaction energy between the surfaces decays slowly at large separation like a double power-law function, *c*_1_′*h*^–2^ + *c*_2_′*h*^–5^. The powers of the separation *h* are products of the long-range vdW interactions and the precoefficients are products of the short-range Coulombic interactions between molecules alongside additional contributions from enthalpy and vdW interactions. Consequently, we obtained an alternative functional form to the empirical uniexponential^37^ and biexponential^20,38^ decay rules for solvation forces. In particular, the biexponential empirical decay rule is a reminiscent of our bipower law analytical result.

At small separations, solvent molecules are expelled from the gap between the two interacting surfaces, leading to an attractive depletion force. In the bubble case, the water density in the film is the same as in the vapor bulk, indicating that the bubbles have coalesced, forming a uniform vapor phase. In the solid particles case, this depletion indicates that the surfaces dry below a certain separation. Evans and co-workers^39^ conducted many studies on the hydrophobic force in terms of drying of surfaces. They used binding potential analysis, confirmed by DFT and grand canonical Monte Carlo (GCMC) simulations to investigate density depletion. They observe depleted regions to arise upon increasing the hydrophobic solute size. In the limit of large solutes the depletion region is extended and oscillations due to packing effects are extremely damped, leading to smooth density profiles. This indicates that our choice of a mean-field treatment, that produces similarly smooth densities, should not have a significant impact on the study of large bodies. Upon increasing the solute–solvent attraction, their GCMC simulations show the depletion layer’s thickness to be reduced around a hydrophobic particle, as also confirmed by their DFT calculations.^40^ This is also the case in our model for solid particles, where, however, we study the case of two interacting surfaces. In fact, from Figure 3 it is evident that larger values of the relative Hamaker constant *H* cause the complete depletion of the film, and therefore the steep attraction, to occur at shorter distances. Their DFT calculations^41^ also highlight that next to very hydrophobic surfaces the depletion layer is approximately 1–2 molecular diameters thick and increases with temperature. The first observation explains why in our force profiles in the bubble case the depletion occurs at surface to surface separations between 2 and 3 molecular diameters—0.6–0.9 nm, as a consequence of the presence of one layer per surface. In the solid case the depletion is at larger distances because of the additional 1 molecular diameter minimum distance between water and wall molecules, but the increase of the depletion zone with temperature is evident—see Figure 3. They come to the conclusion^42^ that, while depleted density is often considered a result of the disruption of the hydrogen-bond network, the fact that it is observed for a simple LJ liquid indicates that it is not related to hydrogen bonds. As a consequence, depletion and the consequent strong hydrophobic attraction are expected for simple liquids—like our vdW model—whenever the solvent is close to phase coexistence and the attraction to the surface is weak. Both these conditions are satisfied by the present study.

A strong attractive force between large hydrophobic bodies becomes visible also in Lum et al.^43^ starting at distances of 10 nm, while liquid water becomes unstable around 5 nm in favor of vapor, leading to drying. This is in agreement with the jump-in distance of surface force experiments. In this work a DFT approach is adopted to study a liquid with a vdW equation of state. DFT calculations are carried out in a mean-field approximation, which can model drying, but not density oscillations. These are retrieved at small scale using a solvent-specific Gaussian distribution depending on the molecular size. While using an approach that is similar to ours and even accounting in the aforementioned way for correlations due to excluded volume, this theory does not provide an analytic expression for the force decay. However, the phenomenology of short-range attraction is qualitatively similar to ours.

In our treatment, wherever vdW attraction between solvent molecules dominates the interaction, the solvation interaction is purely attractive, i.e., a purely hydrophobic interaction. However, should vdW attraction between the molecules in interacting solids and the intervening solvent be greater than the vdW attraction between the solvent molecules themselves, one may observe repulsive, hydrophilic-like, interaction at certain separations that yields repulsive peaks of the interaction energy. Finally, the magnitude of the force obtained in our analysis falls in the range of hydrophobic forces typically measured in experiments.

The present theory applies to hydrophobic particles, where the solvent is not particularly structured next to the surfaces. In this case the interaction is determined by the presence of a depletion layer at short distances and by the slow decay of the single-interface density profile at large distances. Finite-volume effects are accounted for through the volume term *b* of the vdW equation of state and by taking the mean-field density variation integral outside the molecular volume in eq 1, rather than by the structural excess free energy of excluded-volume interactions, e.g., a Carnahan–Starling approximation. Therefore, no correlations due to excluded volume are present. In following work, the model can be expanded to account for hydrophilic interactions by adding polar groups at the particle surfaces. Moreover, the structural free energy of excluded-volume interactions could be included explicitly in the excess Helmholtz free energy, for which a different approximation needs to be used for the DFT due to the formation of structured solvent layers in this case.

## Supporting Information Available

Analytical derivation of the asymptotic expansion for the solvation force across a vapor/liquid/vapor film and expression for a liquid film between solid walls (PDF)
