# Supplementary material for: Solvation Forces Near Hydrophobic Surfaces: A Classical Density Functional Theory Study
Source: J Phys Chem B. 2024 Jul 19;128(30):7457–66. doi: 10.1021/acs.jpcb.4c01426 (PMC11301688; doi:10.1021/acs.jpcb.4c01426)
Supplement: Supplementary file 1 — jp4c01426_si_002.pdf [file jp4c01426_si_002.pdf]

Solvation Forces Near Hydrophobic Surfaces: A Classical Density Functional Theory Study

Simone Riva* and Ofer Manor*

Department of Chemical Engineering, Technion - Israel Institute of Technology, Haifa, 3200003, Israel

E-mail: simone.riva@campus.technion.ac.il; manoro@technion.ac.il

Supporting Information

Asymptotic expansion of the disjoining pressure for bubbles

We derive here the asymptotic expansion for the disjoining pressure between two bubbles. Some approximations are introduced, most noticeably the sharp-interface approximation and the expression of the density profile as a superposition of single-interface densities.

First we define the solution of Eq. (3) in the main text in the presence of a single vapor-liquid interface at $z = 0$ as $\rho_0(z)$. This solution is a sigmoid function also found by Pismen.[1,2] Its horizontal asymptotes at $-\infty$ and $+\infty$ are respectively the vapor and liquid bulk density, ρ_v and ρ_l. These are the two minima of the free energy function $\rho[f(\rho) - \mu]$, in which μ must be such that the minima have equal depth.

In the case of two interfaces at $z = 0$ and $z = h$, we assume the following form of the density, which satisfies the symmetry requirement:

$$\rho(z; h) = \frac{1}{\rho_l}\rho_0(z)\rho_0(h - z). \tag{S1}$$

Although the specific shape of the density profile in our asymptotic analysis depends on this assumption, the general conclusions we will derive do not; the same asymptotics is found using a linear superposition of two single-interface densities, for instance. Moreover, in the limit $h >> \sigma$, the interface region where the density variation occurs will be relatively small, so that it is reasonable to make use of the sharp-interface approximation: $\rho_0'(z) = \rho_l \delta(z)$, as the vapor bulk density ρ_v can be neglected compared to the liquid density ρ_l.

The asymptotic solution of Eq. (3) for $z \to \infty$, thus $\rho_0(z) \to \rho_l$, is found by Taylor-expanding the first term around $\rho_0 = \rho_l$, remembering that $g(\rho_l) - \mu = 0$, since ρ_l is a stationary point of $\rho[f(\rho) - \mu]$, and calculating the integral using a Heaviside-theta shape for ρ_0 inside the integral (sharp-interface approximation):

$$\rho_0(z) = \rho_l - \frac{\pi C_{11}\rho_l}{6g'(\rho_l)} z^{-3} - \frac{g''(\rho_l)}{72 g'^3(\rho_l)} (\pi C_{11}\rho_l)^2 z^{-6}. \tag{S2}$$

Therefore, the single-interface solution for $z \to \infty$ approaches ρ_l with a power-law decay, where the leading term is of order z^{-3} and the first correction z^{-6}. The way the integral has been approximated introduces an error of order z^{-6}, so that it is necessary to add a correction to the corresponding coefficient, which was evaluated numerically. With this correction Eq. (S2) fits accurately the numerical solution.

Differentiating Eq. (2) in the main text with respect to h gives the disjoining pressure of the film. In the sharp-interface approximation,

$$\Pi(h) = -\frac{d\Omega(h)/A}{dh} = -g(\rho(h))\rho_0(h) + \mu\rho_0(h) - \int_{-\infty}^{+\infty} d\zeta\, Q(\zeta)\,[\rho(h-\zeta) - \rho(h)]\,\rho_0(h), \tag{S2}$$

where the integral can be computed by parts. Based on the previous expansion of ρ_0 it is possible to determine the large-h asymptotics. A contribution from the integral term similar to what was found by Pismen[2] in the case of a vanishing Hamaker constant for bubble-liquid

dispersion interactions,

$$\Pi(h) = -\frac{1}{6}\pi C_{11}\rho_l^2 h^{-3} + \frac{(\pi C_{11}\rho_l)^2}{18 g'(\rho_l)} h^{-6}, \tag{S2}$$

is obtained under two assumptions. The first one is that $\rho(h) = \frac{1}{2}\rho_0(h)$, namely that the density at the right interface is a half of the corresponding single-interface density; secondly that ρ_v is negligible.

If one renounces these simplifications, evaluating the correct density at the surface, $\rho(h)$, by numerical integration, and takes into account the first two terms of Eq. (S2) as well, the following expression needs to be added to the disjoining pressure, which becomes considerably more complicated, although it maintains the same form:

$$\begin{aligned}
&\frac{4}{3}\pi C_{11}\sigma^{-3}\rho_v\rho_l - \frac{4}{3}\pi C_{11}\sigma^{-3}\left(\frac{\rho(h)}{\rho_0(h)} - \frac{1}{2}\right)\rho_l^2 - g(\rho_0(0))\rho_l + \mu\rho_l \\
&+ \left[-\frac{4}{3}\pi C_{11}\sigma^{-3}\frac{\rho_v}{\rho_l g'(\rho_l)} + \left(\frac{\rho(h)}{\rho_0(h)} - \frac{1}{2}\right)\frac{8}{3}\frac{\pi C_{11}\sigma^{-3}}{g'(\rho_l)} \right. \\
&\left. + \frac{g(\rho_0(0)) + g'(\rho_0(0))\rho_0(0)}{\rho_l g'(\rho_l)} - \frac{\mu}{\rho_l g'(\rho_l)}\right]\frac{1}{6}\pi C_{11}\rho_l^2 h^{-3} \\
&+ \left[-\frac{g''(\rho_l)}{g'^2(\rho_l)}\frac{1}{3}\pi C_{11}\sigma^{-3}\rho_v - \left(\frac{\rho(h)}{\rho_0(h)} - \frac{1}{2}\right)\frac{2}{3}\frac{\pi C_{11}\sigma^{-3}}{g'(\rho_l)}\left(1 - \frac{\rho_l g''(\rho_l)}{g'(\rho_l)}\right) + \frac{1}{4}\frac{g''(\rho_l)g(\rho_0(0))}{g'^2(\rho_l)} \right. \\
&\left. + \frac{1}{4}\frac{g''(\rho_l)\rho_0(0)g'(\rho_0(0))}{g'^2(\rho_l)} - \frac{\rho_0(0)g'(\rho_0(0))}{2\rho_l g'(\rho_l)} - \frac{1}{4}\frac{\rho_0^2(0)g''(\rho_0(0))}{\rho_l g'(\rho_l)} - \frac{\mu}{4}\frac{g''(\rho_l)}{g'^2(\rho_l)}\right]\frac{(\pi C_{11}\rho_l)^2}{18 g'(\rho_l)} h^{-6}.
\end{aligned} \tag{S3}$$

Here the coefficients of the previous h^{-3} and h^{-6} terms have been isolated, so that the expressions between the brackets are the relative variations of the coefficients. The disjoining

pressure can be written synthetically as

$$
\begin{aligned}
\Pi(h) &= \Pi_0 + c_1 h^{-3} + c_2 h^{-6}, \qquad \text{(S3)}\\
\Pi_0 &= \frac{4}{3}\pi C_{11}\sigma^{-3}\rho_v\rho_l - \frac{4}{3}\pi C_{11}\sigma^{-3}\left(\frac{\rho(h)}{\rho_0(h)} - \frac{1}{2}\right)\rho_l^2 - g(\rho_0(0))\rho_l + \mu\rho_l,\\
c_1 &= \left[-1 - \frac{4}{3}\pi C_{11}\sigma^{-3}\frac{\rho_v}{\rho_l g'(\rho_l)} + \left(\frac{\rho(h)}{\rho_0(h)} - \frac{1}{2}\right)\frac{8}{3}\frac{\pi C_{11}\sigma^{-3}}{g'(\rho_l)}\right.\\
&\quad \left. + \frac{g(\rho_0(0)) + g'(\rho_0(0))\rho_0(0)}{\rho_l g'(\rho_l)} - \frac{\mu}{\rho_l g'(\rho_l)}\right]\frac{1}{6}\pi C_{11}\rho_l^2,\\
c_2 &= \left[1 - \frac{g''(\rho_l)}{g'^2(\rho_l)}\frac{1}{3}\pi C_{11}\sigma^{-3}\rho_v - \left(\frac{\rho(h)}{\rho_0(h)} - \frac{1}{2}\right)\frac{2}{3}\frac{\pi C_{11}\sigma^{-3}}{g'(\rho_l)}\right.\\
&\quad \left(1 - \frac{\rho_l g''(\rho_l)}{g'(\rho_l)}\right) + \frac{1}{4}\frac{g''(\rho_l)g(\rho_0(0))}{g'^2(\rho_l)} + \frac{1}{4}\frac{g''(\rho_l)\rho_0(0)g'(\rho_0(0))}{g'^2(\rho_l)}\\
&\quad \left. - \frac{\rho_0(0)g'(\rho_0(0))}{2\rho_l g'(\rho_l)} - \frac{1}{4}\frac{\rho_0^2(0)g''(\rho_0(0))}{\rho_l g'(\rho_l)} - \frac{\mu}{4}\frac{g''(\rho_l)}{g'^2(\rho_l)}\right]\frac{(\pi C_{11}\rho_l)^2}{18 g'(\rho_l)}.
\end{aligned}
$$

The analytical expressions for g, g' and g'' are easily found differentiating $\rho\, f(\rho)$; $\rho(h)$, the density at the nominal interface , $\rho_0(0)$ and $\rho_0(h)$ are determined numerically. The constant pressure term, Π_0, is negligible, therefore we omit it in our analysis.

Finally, based on the previous definition of the disjoining pressure, $\Pi(h) = -\frac{d\Omega(h)/A}{dh}$, the last equation can be integrated to find the energy per unit area between planar interfaces:

$$
\Omega(h)/A = \frac{c_1}{2}h^{-2} + \frac{c_2}{5}h^{-5} = c_1' h^{-2} + c_2' h^{-5}. \qquad \text{(S4)}
$$

Asymptotic expansion of the disjoining pressure for solid particles

Following a similar derivation it is possible to obtain the analytical asymptotic expansion for the disjoining pressure between two solid surfaces as well. The procedure is complicated by the dependence of Eq. (7) on h both through the integration limits and the parametrical dependence of $\rho(z;h)$. This causes the regular derivative to have twice as many terms as in

vapor-liquid surfaces. The final expression reads

$$
\begin{aligned}
\Pi(h) &= \Pi_0 + c_1 h^{-3} + c_2 h^{-6}, \qquad \text{(S4)}\\
\Pi_0 &= -\rho_0(0)[f(\rho_0(0)) - \mu] - \frac{1}{3}\pi C_{11}\sigma^{-3}\rho_l^2\left[\alpha_s\beta_s - \frac{\rho_0(0)}{\rho_l}\right]\\
&+\frac{2}{3}\pi C_{11}\sigma^{-3}\rho_l^2\left[\alpha_s\beta_s\left(\frac{1}{2}+\frac{\rho_0(0)}{\rho_l}\right) - \frac{\rho_0(0)}{\rho_l}\left(\frac{1}{2}+\frac{1}{2}\frac{\rho_0(0)}{\rho_l}\right)\right],\\
c_1 &= \left[-\frac{\frac{1}{2}[g(\rho_0(0)) - \mu + \rho_0(0)g'(\rho_0(0))] + \frac{\rho_0(0)}{\rho_l}[f(\rho_0(0)) - \mu + \rho_0(0)f'(\rho_0(0))]}{\rho_l g'(\rho_l)}\right.\\
&+\frac{1}{3}\frac{\pi C_{11}\sigma^{-3}}{g'(\rho_l)} - \frac{1}{4(\alpha_s\beta_s - 1)} + \frac{\alpha_s\beta_s}{\alpha_s\beta_s - 1}\left(\frac{1}{2}+\frac{\rho_0(0)}{\rho_l}\right) - \frac{\rho_0(0)}{\rho_l(\alpha_s\beta_s - 1)}\\
&\left.\left(\frac{1}{2}+\frac{1}{2}\frac{\rho_0(0)}{\rho_l}\right) + \frac{2}{3}\pi C_{11}\sigma^{-3}\frac{\alpha_s\beta_s}{g'(\rho_l)}\left(\frac{1}{2}+\frac{\rho_0(0)}{\rho_l}\right) - \frac{2}{3}\pi C_{11}\sigma^{-3}\frac{\rho_0(0)}{\rho_l g'(\rho_l)}\left(1+\frac{\rho_0(0)}{\rho_l}\right)\right]\\
&(\alpha_s\beta_s - 1)\frac{1}{6}\pi C_{11}\rho_l^2,\\
c_2 &= \left[\frac{1}{4}g''(\rho_l)\frac{\frac{1}{2}[g(\rho_0(0)) - \mu + \rho_0(0)g'(\rho_0(0))] + \frac{\rho_0(0)}{\rho_l}[f(\rho_0(0)) - \mu + \rho_0(0)f'(\rho_0(0))]}{g'^2(\rho_l)}\right.\\
&-\frac{1}{4}\rho_0(0)\frac{\frac{1}{2}[2g'(\rho_0(0)) + \rho_0(0)g''(\rho_0(0))] + \frac{\rho_0(0)}{\rho_l}[2f'(\rho_0(0)) + \rho_0(0)f''(\rho_0(0))]}{\rho_l g'(\rho_l)}\\
&+\frac{\pi C_{11}\sigma^{-3}}{12g'(\rho_l)}\left(1 - \frac{\rho_l g''(\rho_l)}{g'(\rho_l)}\right) - \frac{1}{4(\alpha_s\beta_s - 1)} + \frac{\alpha_s\beta_s}{2(\alpha_s\beta_s - 1)}\left(\frac{1}{2}+\frac{\rho_0(0)}{\rho_l}\right)\\
&+\frac{1}{2(\alpha_s\beta_s - 1)}\frac{\rho_0(0)}{\rho_l}\left(1+\frac{\rho_0(0)}{\rho_l}\right) - \frac{1}{6}\pi C_{11}\sigma^{-3}\frac{\alpha_s\beta_s\rho_l g''(\rho_l)}{g'^2(\rho_l)}\left(\frac{1}{2}+\frac{\rho_0(0)}{\rho_l}\right)\\
&\left.+\frac{1}{6}\pi C_{11}\sigma^{-3}\left(\frac{\rho_0(0)g''(\rho_l)}{g'^2(\rho_l)} - \frac{\rho_0(0)}{\rho_l g'(\rho_l)}\right)\left(1+\frac{\rho_0(0)}{\rho_l}\right)\right]\\
&(\alpha_s\beta_s - 1)^2\frac{(\pi C_{11}\rho_l)^2}{18g'(\rho_l)}h^{-6}.
\end{aligned}
$$

Here all quantities have been introduced before, with the exception of α_s and β_s. These are respectively the ratios between the constant C for solid-water and water-water interactions and between the solid and water densities. They are thus defined by: $C_{12} = \alpha_s C_{11}$, $\rho_s = \beta_s \rho_l$. Since the Hamaker constant of solid-water interactions depends on their product, $A_{12} = \pi^2 C_{12}\rho_l\rho_s = \alpha_s\beta_s\pi^2 C_{11}\rho_l^2 = \alpha_s\beta_s A_{11}$, only the latter enters the expansion. As illustrated in the main text, this product, also named $H = \alpha_s\beta_s$, is relevant in our calculation, which were performed for $H = 1.1$, $H = 0.5$ and $H = 0$. The occurrence of the term $(\alpha_s\beta_s - 1)$ reveals

why 1 is a watershed value for the parameter H. Notice that the first and second derivative of f, other than the derivatives of g, are required in this case to calculate the coefficients c_1 and c_2.

The interaction energy can be calculated again by integrating the disjoining pressure:

$$\Omega(h)/A = \frac{c_1}{2}h^{-2} + \frac{c_2}{5}h^{-5} = c_1'h^{-2} + c_2'h^{-5}. \tag{S6}$$

In this case, the linear term $-\Pi_0 h$ would represent the energy gain due to extensivity of the Landau free energy. Therefore it was removed, to avoid accounting for energy contributions due to sheer addition of particles as a consequence of the film becoming thicker and not related to solvation. It was also subtracted from the solution of numerical computations, where $-\Pi_0$ was calculated as the bulk energy per unit volume (pressure): $-\Pi_0 = \rho_l\,[f(\rho_l) - \mu]$.

References

(1) Pismen, L.; Pomeau, Y. Disjoining Potential and Spreading of Thin Liquid Layers in the Diffuse Interface Model Coupled to Hydrodynamics. *Physical review. E, Statistical physics, plasmas, fluids, and related interdisciplinary topics* **2000**, *62*, 2480.

(2) Pismen, L. Nonlocal diffuse interface theory of thin films and the moving contact line. *Physical review. E, Statistical, nonlinear, and soft matter physics* **2001**, *64*, 021603.
